# COVID-19 Autopsy in India: Protocols, Procedures, and Experiences

**DOI:** 10.7759/cureus.18984

**Published:** 2021-10-22

**Authors:** Jayanthi Yadav, Brinda Patel, Mahaluxmi S, Sravan JS

**Affiliations:** 1 Forensic Medicine, All India Institute of Medical Sciences, Bhopal, Bhopal, IND

**Keywords:** covid autopsy in developing nation, autopsy in infected case, precautions in covid-19 autopsy, post-mortem on covid-19 deaths, methodology of covid-19 autopsy, covid-19 sample collection from autopsy, covid-19 autopsy, - autopsy protocol in covid-19 deaths

## Abstract

Background: Coronavirus disease 2019 (COVID-19) has besieged mankind because of its novelty, causing a global health crisis. The autopsy-based studies provide a crucial role in understanding the pathophysiology and the behavior of the disease. But there is a paucity of such studies in the world especially so from developing nations. Conducting a complete autopsy on infectious bodies like COVID-19 requires conducive infrastructural setup and protocols suited to the needs, and precautions are to be taken meticulously.

Methods: A complete pathological autopsy was conducted on a known case of a COVID-19-hospitalized patient, who died in our institution, with the aim to look for histopathological changes in each organ and to compare these findings with clinical findings such as duration of hospitalization, mechanical ventilation, comorbidities, biochemical parameters, and the result of real-time* polymerase chain reaction* (RT-PCR) of the tissues. The complete autopsy was performed after obtaining consent from the family, and the study was approved by the Institutional Ethics Committee. Histopathological examination (HPE) and RT-PCR were conducted on the tissue collected during autopsy. Clinical and biomedical data were collected and correlated.

Result:* *The written informed consent from the family could be obtained in only 15.3% of cases, which was a limiting factor. The post-mortem interval ranged from 3.5 to 19.5 hours*. *The gross findings revealed pathologic features of viral infection as well as existing comorbidities in all the organs. The development of protocols and new innovations to limit the spread of infection, taking into consideration the limited facilities, which are described in this article, resulted in the successful completion of all the autopsies with a good sample collection, and nobody in the autopsy team was tested positive for severe acute respiratory syndrome coronavirus 2 (SARS-CoV-2).

Conclusions:* *The experience gained from these 21 COVID-19 autopsies helps to outline the basic or minimal requirements for conducting autopsies in highly infectious cases even in not-so-ideal conditions and also provides guidelines to be used while conducting such autopsies, especially in developing countries.

## Introduction

Severe acute respiratory syndrome coronavirus-2 (SARS-CoV-2) is a virus responsible for coronavirus disease 2019 (COVID-19), which originated in Wuhan, China, around December 31, 2019, and has subsequently spread across the globe.

The first confirmed case of COVID-19 in India was reported on January 30, 2020, in Kerala [1]. It gradually started spreading all over the country, and the peak was reached in mid-September during the first wave [2].

With the emergence of any new infectious disease, an autopsy is considered to be the gold standard to know about the extent, spread, and pathophysiology of the disease. In the context of limited scientific knowledge and evidence of SARS-CoV-2 infection, it is becoming increasingly necessary for post-mortem investigations to be performed on COVID-19 cases [3]. But performing autopsies in infectious cases possesses an inherent risk to the autopsy team that acts as a deterrent to know more about such disease entities. The majority of such studies related to COVID-19 were conducted in developed nations of Europe [4-6] and the United States of America (USA) [7].

With the actual spread and mortality due to COVID-19 in India turning out to be quite different during the first wave from what was projected [8], the necessity to carry out an autopsy-based study was felt to know more about the behavior of this virus in the Indian population.

Dealing with infectious autopsies and the available facilities at our mortuary required us to change our operational autopsy protocol, maintain all necessary precautions, and also obtain qualitative technical results that can be useful in understanding this novel disease.

We report in this article our experience and innovations adapted in a resource-limited setting of a developing nation while adopting the standard protocols for autopsy in the first series of complete COVID-19 autopsies conducted in India. The scenario, limitations, modifications, and technical details of conducting autopsies of such highly infectious cases in developing countries like India are discussed and compared with techniques mentioned in other similar studies from the more developed Western countries.

## Materials and methods

When the idea of performing complete autopsies on COVID-19 decedents was conceived, the authors meticulously studied the recommendations provided by the Center for Disease Control and Prevention (CDC) [9], Royal College of Physicians (RCP) [10], COVID-19: Guidelines on Dead Body Management[11], and World Health Organization (WHO) [12] for performing autopsies on highly infectious cases. The published autopsy studies specifying the technical details were also referred [3,7,13], and a protocol relevant to the working conditions of the mortuary at our institute was developed.

As per Indian Council of Medical Research (ICMR) guidelines, routine autopsies on COVID-19-positive cases are not recommended. Hence, the autopsies were conducted as part of the research project as a special case after prior approval from the Institutional Ethics Committee of our institution. All cases who tested positive by real-time polymerase chain reaction (RT-PCR) of nasopharyngeal and/or oropharyngeal swab samples got admitted at our institute and consecutively died during treatment between August 5 and October 23, 2020, and those RT-PCR-positive patients who were brought dead, in which consent from family could be obtained, were considered as potential subjects for the study.

**Figure 1:**
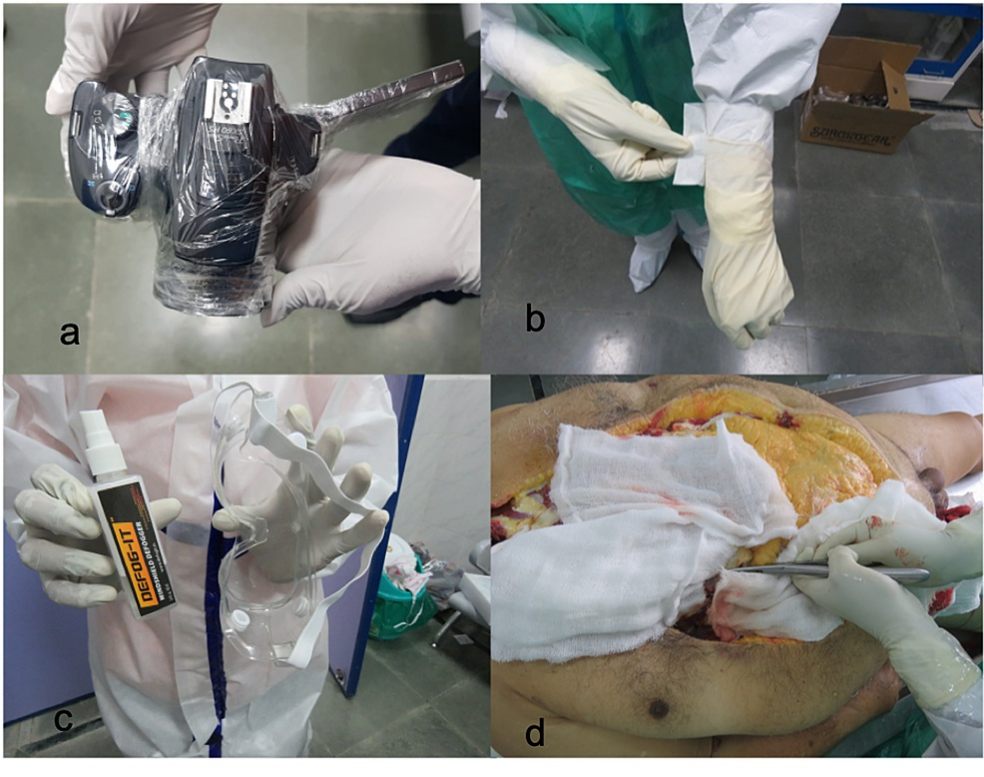
Modifications adopted during autopsy: (a) Wrapping of the camera with cling film to minimize contamination; (b) securing the outer gloves with micropore tape with its end folded for easy doffing; (c) spraying of antifogging spray to avoid fogging inside goggles; and (d) covering the surrounding areas around the cut to minimize aerosol transmission

The dead body was received in the mortuary after being prepared as per the guidelines issued by the COVID-19: Guidelines on Dead Body Management [11]. The movement in and out of the autopsy room during the autopsy was restricted [14] by ensuring the availability of all instruments (scalpel, forceps, knives, trays, syringes, camera, etc.), formats for data entry, 1% sodium hypochlorite, preservative solutions (10% neutral buffered formalin, normal saline, rectified spirit, etc.) inside the autopsy room. Extra pairs of gloves, knives, containers, and vials were also kept ready in case of need. All containers and viral transport medium (VTM) vials required for sample collection were labeled prior to the start of the autopsy. The clinical notes of the deceased were well studied beforehand. The camera and weighing machine were wrapped with plastic cling wrap to avoid soiling and contamination of equipment. The cling wrap was discarded in the autopsy room itself after completion of the autopsy (Figure 1, Panel a). The packing materials (boxes, plastic bags, cling wrap, etc.) required for sample preservation were also kept handy. A checklist of pre-autopsy preparations was looked at before entering the autopsy room so as not to miss out on anything needful.

The autopsies were conducted wearing full personal protective equipment (PPE) that included an N95 mask, caps, double gloves, shoe cover, gumboots, gown/overall, plastic apron, and goggles. The donning and doffing were done as per the recommended protocol [15]. Special care was taken especially during doffing to avoid contamination [16].

A few modifications were made to suit our needs, which included: (i) outermost gloves secured in place using micropore tape and their loose ends being folded for ease of doffing (Figure 1, Panel b). This was done to prevent slippage during the autopsy. (ii) To overcome the problem of fogging of goggles (especially during humid days), its inner surface was sprayed with the antifogging solution used in a car (Figure 1, Panel c).

The autopsy was performed in an isolated autopsy room equipped with heating ventilation and air conditioning (HVAC) having 12 air cycle changes per hour (ACH) and high-efficiency particulate air (HEPA) filters. Separate zones (clean, buffer, and dirty) were maintained inside the autopsy room to limit the spread of infection.

The autopsy team was constant for all the autopsies and comprised of four forensic medicine doctors with pre-defined roles: one senior faculty, one senior resident, and two junior residents. The presence of an experienced person was essential to supervise that Low Aerosol Generation Procedures (AGP) followed during the autopsy. Two doctors including the senior faculty were involved in dissection and collection of samples (dirty area), one for photography and noting down the findings (clean area) and the other one for handling and packaging of samples (buffer area) to minimize the spread of infection.

Low aerosol-generating techniques were employed during the autopsy, which included the usage of hand-held instruments (chisels, hammers, and rib shears) instead of an electric saw; the areas being dissected were covered by gauze to minimize spillage and splashes. Care was taken to open one cavity at a time; 1% sodium hypochlorite solution was sprayed on the body at each step after collection of samples, and the knife and forceps were sterilized with the spirit or a new knife was used before each sampling to prevent contamination.

During the external examination, the nasopharyngeal swab was taken for microbiological analysis. All details such as built, height, presence of petechial hemorrhages, bruises, or any other relevant findings were noted. While turning the body to examine the back, adequate care was taken to cover the mouth and nostrils with gauze.

The cranial cavity was opened first. After reflecting on the scalp, the skull was opened using a hand-held hammer and chisel. The swab was collected immediately after the removal of the dura; 5 mg tissue was collected for microbiological analysis from the left parietal lobe with a sterile knife. The brain was then removed, weighed, and examined for any gross pathology like edema, hemorrhage, thrombosis of sagittal and other veins, swelling, etc. Tissue was then collected for histopathology from the cerebellum, both parietal lobes (cut section at the level of mammillary bodies), both frontal lobes and base of the frontal lobe, olfactory bulb with tract, and circle of Willis. In addition, samples were preserved from regions where any gross abnormality could be identified. After sample collection, the remaining brain was put back in the cranial cavity, the skull cap was replaced, and the scalp was stitched back.

The thoracoabdominal cavity was opened by giving an I-shaped midline incision extending from the chin to pubic symphysis. Virchow’s technique of dissection was followed. A tracheal swab was collected after opening the trachea using a new sterile blade. The ribs were then cut with rib shears, and care was taken to cover the surrounding area of cut with gauze to limit the spread of aerosols (Figure 1, Panel d). In the thoracic cavity, the presence of any pleural or pericardial pathology (adhesions, effusions, etc.) was noted, and samples of pleural or pericardial fluid (5 ml) were collected accordingly in sterile containers. The pericardium was reflected, and the heart was removed and weighed. Coronaries were examined for any occlusion, and samples for histopathology were preserved from walls of each chamber, interventricular septum, AV and SA nodes, and the region from where any gross pathology could be noted. Then each lung was examined in situ, and a swab was collected from each primary bronchus; 5 mg of lung tissue for microbiology was taken from each lower lobe with a sterile knife. Lungs were carefully taken out one by one. Organs were held in place firmly using gauze to prevent slippage and spillage. Each lung was weighed and examined. Tissue from hilar, apical, basal regions and any other areas as required were collected for histopathological analysis. The lungs and heart were then placed back in the thoracic cavity, and the rib cage was replaced.

The abdominal cavity was then opened and examined for the presence of any fluid/blood. If present (5 ml), they were collected accordingly in sterile containers. The liver, spleen, kidneys, pancreas, and uterus (in females) were taken out one by one, weighed, examined, and samples were collected from each of these. The whole pancreas along with adjoining part of the intestine, half of each kidney, piece of spleen and liver, and uterus were preserved for histopathological examination. Swabs from the liver and both kidneys were taken after giving a nick to the tissue with a sterile knife; 5 mg of the liver and kidney tissue were also collected for microbiological analysis.

All organs were then replaced, and thoracoabdominal cavities were stitched back by the persons performing the dissection. The body was then cleaned properly with 1% sodium hypochlorite solution before being packed and placed in a leakproof body bag. All the soiled gauze, cotton, etc. were discarded as per Biomedical Waste Management (BMW) Guidelines for disposal of infectious COVID-19 waste [17].

After completion of the autopsy, the paper on which the findings were recorded was photographed and then discarded before exiting the autopsy room. The outer gown was discarded in the autopsy room in a bin kept four feet away from the autopsy table, and doffing was done outside in a designated doffing area. The clean persons in the autopsy room helped the doffing of the autopsy surgeon. The autopsy room and the equipment used were sanitized using 1% hypochlorite solution after each autopsy.

Samples for histopathological examination (HPE) were collected in 10% neutral buffered formalin-filled plastic containers, which were then covered with plastic cling wrap before covering them with plastic lids. Similarly, samples for microbiological analysis were preserved in VTM vials. The boxes and vials were then kept in a separate plastic bag and transferred to a cardboard box kept outside the autopsy room. From here, they were transported to respective labs at the earliest taking all necessary precautions.

## Results

**Figure 2:**
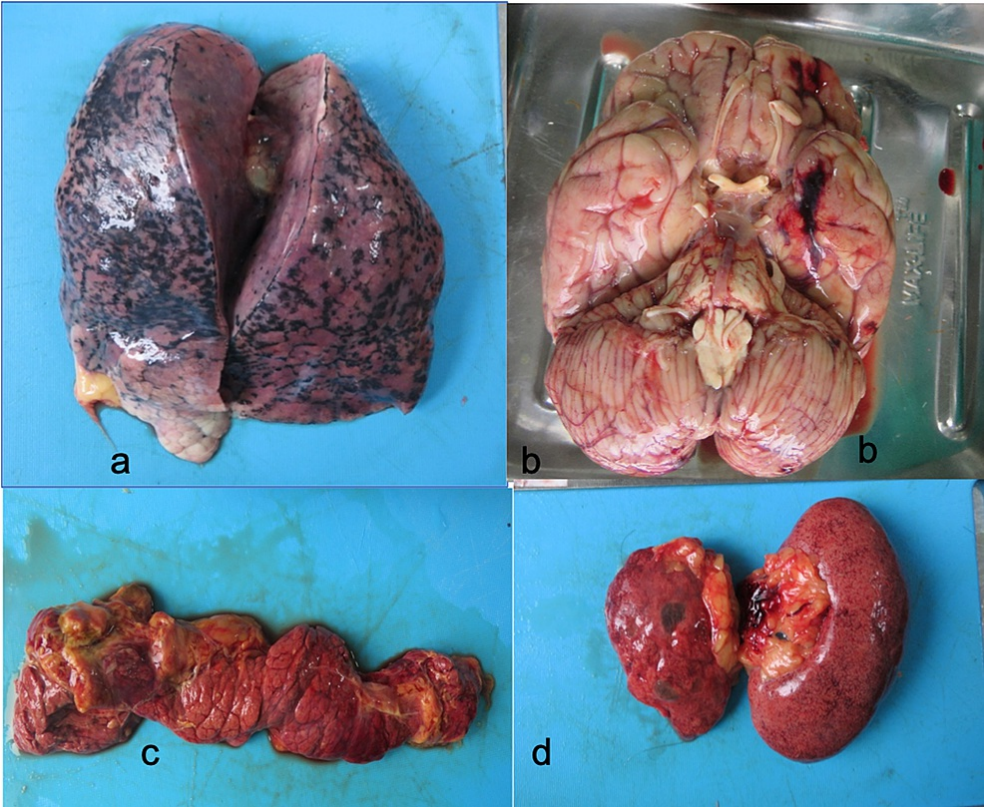
Gross findings of various organs during autopsy: (a) gross examination of lungs showing relatively unremarkable lungs with mild edema and consolidation; (b) gross examination of brain showing edema and subarachnoid hemorrhage (Case No. 04); (c) gross examination of pancreas showing necrotic and hemorrhagic changes; (d) gross examination of kidneys showing contracted and flea-bitten kidneys

During the study period, 137 families of COVID-positive deaths were approached for the consent of which in only 21 cases, consent could be obtained in which complete autopsies were performed. The clinicopathological parameters of these cases are depicted in Table 1. The gross findings were noticed and recorded (Figure 2).

**Table 1:** Clinicopathological characteristics of cases RT-PCR, Real-time polymerase chain reaction.

Age in Years (Range and Mean)	25-84 (60.8)
Sex M:F ratio	15:6
Post-mortem interval (hours)	3.5-19.5 (11.9)
Comorbidities	
Diabetes mellitus	15 (71.4%)
Obesity	6 (28.5%)
Hypertension	13 (61.9%)
Hypothyroidism	4 (19%)
Coronary artery disease	2 (0.09%)
Others	6 (28.5%)
Nasopharyngeal swab RT-PCR positivity	19 (90.4%)
Tracheal swab RT-PCR positivity	18 (85.7%)
Consent (out of 137 cases)	15.3%

## Discussion

The execution of an autopsy in a patient who died of SARS-CoV-2 presents a lot of challenges to the personnel carrying out the procedure. Among these, the most important ones are of minimizing the risk of infection in the autopsy personnel and preventing the spread of the virus outside the autopsy room. Another is the need to carry out the autopsy as soon as possible after death and perform it quickly in order to have as little tissue damage as possible from post-mortem autolysis [13]. This is a rare report of complete autopsies on COVID-positive deaths from India.

In our experience, the biggest obstacle in conducting these autopsies was obtaining consent from the family of the deceased. In this study, the ratio of obtaining consent was nearly 15% (21 out of 137 cases). This was a global issue, except in certain situations when consent can be imposed as in Germany where the autopsy orders for all COVID-19 deaths were issued by Hamburg public health authorities in accordance with the German Infection Protection Act [5] and in a study by Youd et al. [4] who autopsied nine cases, where all autopsies were conducted with the intention of finding the cause of death on orders of the coroner, hence consent was not required.

In India, the guidelines for dead body management require that the dead body of a COVID-19 deceased not be handed over to the family but be cremated/buried by the municipality. Despite the fact that the family would not be able to take the body home for performing last rites, consent was still not forthcoming due to various reasons like religious beliefs and expected mutilation of the body of their loved ones.

The autopsies were conducted as soon as possible after the death of the patient to minimize autolytic changes and to avoid delay in handing over the dead body. The average time lapsed was 11.9 hours (ranging from 3.5 hours to 19.5 hours). Hence most of the autopsies were conducted at odd hours of the day. The delay was mainly due to the time taken by the family members to reach the mortuary from distant places after intimation of death and give consent for the autopsy.

Going through the clinical details of the deceased was a very important step in knowing about the case. It not only helped in knowing which samples needed to be collected but also which pathological changes could be attributed to chronic illness and which to the treatment. The usefulness of this clinical knowledge has been emphasized by Carpenito et al. [13].

In our experience, the optimum number of persons inside the autopsy room is four. Few studies have reported conducting autopsy with a team of two to three persons [14]. It was observed that four team members limited the autopsy exposure time to almost 1.5-2 hours. A reduced number of persons to less than four not only increased the time taken during the autopsy, which in turn increased the exposure time to the infection, but it also interferes with the maintenance of different zones such as clean, buffer, and dirty zones inside the autopsy room. In India, as in any other country of the subcontinent, pathological autopsies are in general rarely performed. Also, unlike Western countries where forensic autopsies are performed mainly by pathologists, the medicolegal autopsies in India are routinely conducted by forensic experts or any other Registered Medical Practitioners, but they conduct the autopsies only from a medicolegal perspective rather than the pathological aspects. In India, forensic medicine is a separate specialty in post-graduation [18]. Forensic medicine experts are better versed in conducting autopsies because of the sheer number of autopsies carried out by them as compared to the pathologists.

As per the briefing of The Royal College of Pathology, United Kingdom, the Advisory Committee on Dangerous Pathogens (ACDP) within the Health and Safety Executive [HSE] has categorized COVID-19 as an HG-3 (hazard group) infection [10]. The mode of transmission is largely via respiratory droplets. Another possibility of spread by surface contamination is also mentioned by CDC [9]. Guidelines have been provided by these agencies specifying the requirements and precautions to be taken while performing autopsies in such cases.

All these agencies have mentioned the necessity of a negative-pressure autopsy room and a minimum of six ACH to conduct autopsies of highly infectious cases (HG3 organisms) with a downdraft table. The WHO's guidelines suggest Biosafety Level 3 (BSL-3) for autopsies performed on patients who died of SARS-CoV-2 [12,13]. The facilities available at the mortuary of our institute did not have a BSL-3 setup or a downdraft autopsy table as recommended. Here, the facilities correspond with the conditions required for a BSL-2 setup. Mao et al. [16] advise performing autopsies inside specially designed safety bags if BSL-3 facilities are not available, but conducting autopsies within a body bag may restrict the observation as well as hinder the clean collection of samples.

The mortuaries in India are also the most neglected area of any hospital, and in many places, they even lack the basic infrastructure to carry out routine non-infectious autopsies. Therefore, considering the risk involved in conducting such high-risk autopsies, customized protocols need to be developed in each setup keeping in view the recommendations and the facilities available.

The present study highlights the method and modifications adopted to suit our conditions. The authors recommend covering the dissected area with gauze to minimize the aerosol spread and limit the infection. The proper donning and especially doffing of PPE also play an important role in containing the spread and safeguards the autopsy surgeons. The fact that no case has been so far reported of contracting this disease by any autopsy team goes on to validate this fact [19,20].

## Conclusions

COVID-19 pandemic has once again made the world realize the importance of autopsies in gathering crucial information about any novel disease entity. In developing nations like India, performing pathological autopsies of highly infectious cases is quite uncommon and hence is generally avoided due to lack of ideal conditions. The experience gained from these 21 COVID-19 autopsies helps us to outline the basic or minimal requirements for conducting autopsies in highly infectious cases even in not-so-ideal conditions, namely adequate personal protective measures, adequate ventilation with a negative-pressure autopsy room, and low AGP techniques during the autopsy. If these points are considered and meticulously followed, then such infectious autopsies, even in a mortuary with basic facilities, can become a possibility.

## References

[1] Andrews MA, Areekal B, Rajesh KR (2020). First confirmed case of COVID-19 infection in India: a case report. Indian J Med Res.

[2] (2021). Coronavirus: has the pandemic really peaked in India?. https://www.bbc.com/news/world-asia-india-54596707.

[3] Santurro A, Scopetti M, D'Errico S, Fineschi V (2020). A technical report from the Italian SARS-CoV-2 outbreak. Postmortem sampling and autopsy investigation in cases of suspected or probable COVID-19. Forensic Sci Med Pathol.

[4] Youd E, Moore L (2020). COVID-19 autopsy in people who died in community settings: the first series. J Clin Pathol.

[5] Edler C, Schröder AN, Aepfelbacher M (2020). Dying with SARS-CoV-2 infection-an autopsy study of the first consecutive 80 cases in Hamburg, Germany. Int J Legal Med.

[6] Menter T, Haslbauer JD, Nienhold R (2020). Postmortem examination of COVID-19 patients reveals diffuse alveolar damage with severe capillary congestion and variegated findings in lungs and other organs suggesting vascular dysfunction. Histopathology.

[7] Barton LM, Duval EJ, Stroberg E, Ghosh S, Mukhopadhyay S (2020). COVID-19 autopsies, Oklahoma, USA. Am J Clin Pathol.

[8] Chetterje P (2020). Gaps in India's preparedness for COVID-19 control. Lancet Infect Dis.

[9] (2020). Collection and submission of postmortem specimens from deceased persons with confirmed or suspected COVID-19. https://www.cdc.gov/coronavirus/2019-ncov/hcp/guidance-postmortem-specimens.html.

[10] Osborn M, Lucas S, Stewart R, Swift B, Youd E (2020). Autopsy practice relating to possible cases of COVID-19 (2019-nCov, novel coronavirus from China 2019/2020). https://www.rcpath.org/uploads/assets/d5e28baf-5789-4b0f-acecfe370eee6223/fe8fa85a-f004-4a0c-81ee4b2b9cd12cbf/Briefing-on-COVID-19-autopsy-Feb-2020.pdf.

[11] Government of India Ministry of Health & Family Welfare Directorate General of Health Services (EMR Division) (2020). COVID-19: Guidelines on Dead Body Management. https://www.mohfw.gov.in/pdf/1584423700568_COVID19GuidelinesonDeadbodymanagement.pdf.

[12] (2020). WHO post-outbreak biosafety guidelines for handling of SARS-CoV specimens and cultures. https://www.who.int/publications/i/item/infection-prevention-and-control-for-the-safe-management-of-a-dead-body-in-the-context-of-covid-19-interim-guidance.

[13] Carpenito L, D'Ercole M, Porta F (2020). The autopsy at the time of SARS-CoV-2: protocol and lessons. Ann Diagn Pathol.

[14] Hanley B, Lucas SB, Youd E, Swift B, Osborn M (2020). Autopsy in suspected COVID-19 cases. J Clin Pathol.

[15] (2020). How to put on and how to remove personal protective equipment. https://www.who.int/publications-detail-redirect/WHO-HIS-SDS-2015.1.

[16] Mao D, Zhou N, Zheng D (2020). Guide to forensic pathology practice for death cases related to coronavirus disease 2019 (COVID-19) (trial draft). Forensic Sci Res.

[17] (2020). Guidelines for Handling, Treatment and Disposal of Waste Generated During Treatment/Diagnosis/Quarantine of COVID-19 Patients. https://www.mohfw.gov.in/pdf/63948609501585568987wastesguidelines.pdf.

[18] (2020). P.G. Medical Education Regulations. P.G. Medical Education Regulations.

[19] Sperhake JP (2020). Autopsies of COVID-19 deceased? Absolutely!. Leg Med (Tokyo).

[20] Han B, Bhalla R, da Silva Lameira F, Vander Heide RS, Love GL (2020). COVID-19 autopsies and personal protective equipment. Arch Pathol Lab Med.

